# Imaging ATUM ultrathin section libraries with WaferMapper: a multi-scale approach to EM reconstruction of neural circuits

**DOI:** 10.3389/fncir.2014.00068

**Published:** 2014-06-27

**Authors:** Kenneth J. Hayworth, Josh L. Morgan, Richard Schalek, Daniel R. Berger, David G. C. Hildebrand, Jeff W. Lichtman

**Affiliations:** ^1^Howard Hughes Medical InstituteAshburn, VA, USA; ^2^Department of Molecular and Cell Biology, Harvard UniversityCambridge, MA, USA

**Keywords:** connectomics, ATUM, volume EM, scanning electron microscopy, ultramicrotome, imaging software, tape collection, serial-section electron microscopy

## Abstract

The automated tape-collecting ultramicrotome (ATUM) makes it possible to collect large numbers of ultrathin sections quickly—the equivalent of a petabyte of high resolution images each day. However, even high throughput image acquisition strategies generate images far more slowly (at present ~1 terabyte per day). We therefore developed WaferMapper, a software package that takes a multi-resolution approach to mapping and imaging select regions within a library of ultrathin sections. This automated method selects and directs imaging of corresponding regions within each section of an ultrathin section library (UTSL) that may contain many thousands of sections. Using WaferMapper, it is possible to map thousands of tissue sections at low resolution and target multiple points of interest for high resolution imaging based on anatomical landmarks. The program can also be used to expand previously imaged regions, acquire data under different imaging conditions, or re-image after additional tissue treatments.

## Introduction

The three dimensional (3D) structure of biological tissues can be ascertained at high resolution by cutting plastic-embedded tissue into a series of ultrathin sections, imaging those sections with an electron microscope, and reconstructing the objects contained therein *(volume EM)*. Obtaining such volumetric reconstructions is especially useful for analysis of nervous system samples because nerve cells distribute their processes over extended volumes and only with the resolution of electron microscopy (EM) is it possible to identify the network of synaptic connections between all the neurons. This dense synaptic connectivity data is critical to understanding how nervous systems process information (Morgan and Lichtman, 2013).

Until recently, volume EM required manually collecting a series of ultrathin sections onto the nanometers thin plastic film of a transmission electron microscope (TEM) grid. Because of the thin substrate, the process of making serial sections can be painstaking and is subject to tissue loss that poses a serious challenge for very large volume reconstructions (Gay and Anderson, 1954; Harris et al., 2006). With the introduction of high-performance field emission scanning electron microscopy (SEM) (Joy, 1991; Bogner et al., 2007), high quality images can be acquired from the surface of ultrathin sections thereby removing the need to mount sections on an electron-transmissive substrate. Several new imaging strategies have emerged to take advantage of EM surface imaging to facilitate the production of large EM image volumes.

One strategy that is based on SEM surface imaging is to image the surface of a plastic-embedded block of brain tissue that is mounted directly inside the SEM. Tens of nanometers of the block's top surface are then removed by either a microtome in the serial blockface EM (SBEM) approach or a focused ion beam (FIB) in the FIB-SEM approach to expose a new surface for imaging. This procedure can be repeated many thousands of times to produce a volume EM image set (Denk and Horstmann, 2004; Knott et al., 2008).

**Figure 1:**
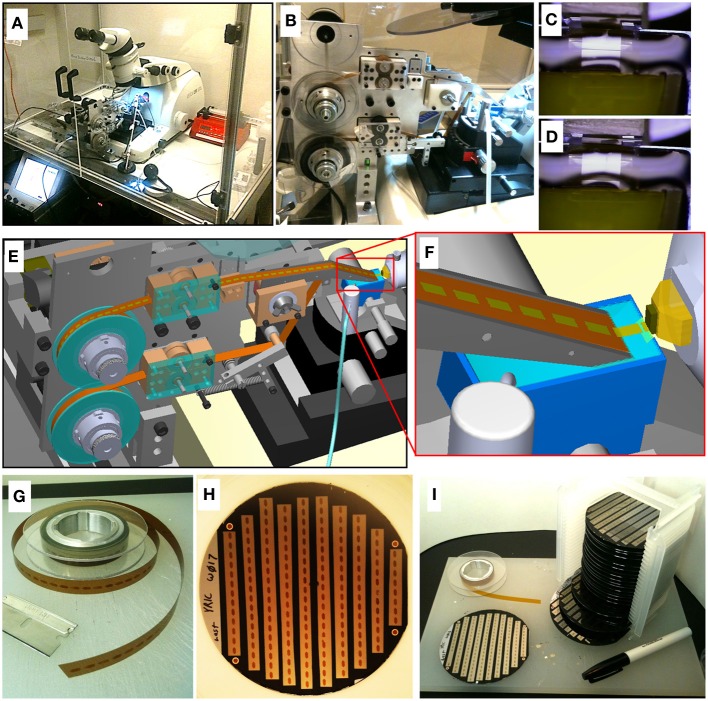
**ATUM-SEM process. (A)** Picture of ATUM tape collection device installed on a commercial ultramicrotome housed in an environmental control chamber. **(B)** Side view of ATUM. **(C,D)** Sequential video images of section collection. **(E)** CAD rendering of ATUM showing path collected sections take from the knife boat to the final take-up reel. **(F)** CAD zoom in on knife boat showing collection process. **(G)** Picture of unraveled tissue-tape on take-up reel containing a series of ultrathin sections. Each dark rectangle is a section. **(H)** Picture of a 100 mm diameter silicon wafer with 10 tissue-tape strips adhered to it. There are a total of 162 ultrathin sections on this one wafer. **(I)** Picture of 20 wafers all filled with tape strips from a single ATUM run consisting of over 15 m of tissue tape. This collection of 20 wafers is a single UltraThin Section Library (UTSL) containing over 3000 ultrathin sections, representing a total tissue volume of over 0.2 mm^3^.

A second strategy, which we adopt in this paper, takes advantage of the surface imaging capabilities of SEM by mounting ultrathin sections on a much more stable substrate than can be used in TEM. In the recently invented Automatic Tape-collecting UltraMicrotome SEM (ATUM-SEM) process (Schalek et al., 2011), the ultrathin sections cut by a commercial ultramicrotome are immediately and automatically collected from the knife's water boat onto the surface of a partially submerged conveyor belt made of sturdy plastic tape (Figure 1). SEM imaging of the series of sections collected on the tape produces a dataset of micrographs which renders individual planes through a 3D tissue volume. This tape collection allows thousands of ultrathin sections to be collected in an automatic way. Because of the uninterrupted flow of tissue onto the relatively wide conveyor belt, the sections obtained can be thinner, larger in area (several square millimeters each), and of higher quality (without tears, etc.) than are obtained by manual collection for TEM.

A major challenge of the ATUM-SEM approach is setting up many thousands of targeted image acquisitions from tissue sections spread across meters of ATUM collection tape. To convert these sections into an image volume, the ATUM's tape must be mounted in the SEM and a region of interest on a section must be positioned beneath the electron beam for imaging. The corresponding region of interest must be found again and again on all subsequent sections. Each section's target region must be positioned, rotated, and focused beneath the electron beam to obtain a high resolution image series. Here we describe a semi-automatic microscope control software package named WaferMapper that can orchestrate all of these steps to produce volume EM image sets from an ATUM tape.

With the proper software solution for handling the additional imaging complexity, the ATUM-SEM process has several potential advantages over alternative techniques. The most obvious advantage over block-face techniques is that the ATUM-SEM technique does not destroy the tissue as it is being imaged. Low resolution images of the entire tissue volume can, therefore, be taken relatively quickly. This overview image volume can be used to plan efficient, targeted high resolution imaging volumes encompassing only those regions crucial to the biological study at hand. Additional high resolution imaging forays on the same sectioned material can be conducted at multiple later times if desired.

The importance of this ability becomes apparent when one considers the time and storage requirements involved in imaging 3D volumes at nanometer resolutions. For example, consider a 0.5 × 2.5 × 3 mm block of mouse brain trimmed to encompass regions of the lateral geniculate nucleus (LGN) and primary visual cortex (V1) along with intact axonal projections connecting the two (MacLean et al., 2006). On the ATUM, such a “visual thalamocortical slice” block could potentially be reduced to a tape of 17,000 × 30 nm thick sections collected with just a few days of sectioning. If imaged in total, with a typical 5 nm in-plane resolution, this volume would require storage of 5 petabytes of image data (17,000 sections each montage imaged with 500,000 × 600,000 pixels). Worse still, if imaged at a standard rate of about 10 megapixels per second, a single microscope would require over 15 years to image the volume, seemingly putting such a study out of reach today. However, the actual connected regions of the LGN and V1 represent only a tiny fraction of the full slice volume which they span. If one could direct high-resolution imaging mainly to those regions which are actually connected then the total imaging time could potentially be reduced by a factor of 10× or more. One way to efficiently direct such high-resolution imaging would be to utilize an iterative process of first making a low resolution image set of the entire volume and then use several passes of directed, medium and high-resolution imaging to narrow in on and eventually high-resolution image only those parts containing an intact thalamocortical circuit. An additional advantage afforded by non-destructive ultrathin section collection is that imaging time can be further reduced by dividing up the ATUM tape so that it can be simultaneously imaged in parallel across multiple SEMs.

This type of parallel, multi-scale, directed-access volume EM imaging—which is not possible in blockface approaches and extremely difficult when handling individual TEM grids—is possible given the large tissue volumes and the robustness to re-imaging and tissue handling of the ATUM-SEM technique. In this paper we use the term “UltraThin Section Library” (UTSL) to describe a collection of many thousand ATUM-collected ultrathin sections which have been securely mounted on wafers for SEM imaging and which have undergone all of the coordinate mapping steps and low resolution overview imaging necessary to allow quick and easy random-access imaging of any point in the volume. These mapping steps are performed through our custom SEM-automation software–WaferMapper.

Our vision is that a researcher using such an UTSL and WaferMapper should be able to quickly browse through the entire tissue volume at low resolution, identify salient anatomical features, and then graphically specify a subvolume for automated imaging. The WaferMapper software then instructs which wafers to load into the SEM leaving the software to automate all subsequent imaging operations.

In order to take full advantage of a large tissue library, the mapping and imaging software must meet the following criteria:

- **Automated imaging**—The first goal of an automated image acquisition software package is to allow a user to image the corresponding region of tissue on all of the sections in a tissue library without having to manually direct the microscope to each section. Ideally, the user should be able to pick a target region within the software, load a wafer, and leave the microscope while the imaging takes place automatically.
- **High throughput**—To reconstruct large regions of tissue at high resolution, images must be acquired quickly. Scan speeds within a single image currently range from about 0.5 to 20 million pixels per second (MPS) using the commercially available SEMs and detectors described in this paper. This large range of imaging speeds reflects the wide range of staining techniques used in connectomics studies as well as differences in the efficiency and bandwidth of different types of detectors used under various imaging conditions. Ideally the acquisition overhead (time spent between image scanning) should be less than the actual image acquisition time. Eliminating human involvement in the image acquisition procedure is an important part of reducing overhead. In addition, however, automated steps such as stage movements, focusing, and image retakes also need to be accomplished quickly so as not to slow down the throughput. (We provide a table breaking down actual data acquisition times for key WaferMapper steps in the Example data section below).
- **Robustness**—The software must allow for variations in tissue properties, as well as staining, cutting, and imaging conditions. In particular, the software must be able to find and image the corresponding region in serial sections that may appear different due to staining artifacts, damage during cutting, or biological changes in the tissue. If changes in the tissue or the microscope result in failures to target the correct tissue region or acquire high quality images, these failures should be detected and corrected without the requirement of human intervention.

WaferMapper has been designed to meet these three goals. Its central strategy is to first map the dataset using low resolution imaging so that the time consuming process of high resolution imaging can be intelligently targeted and automatically executed. In addition, WaferMapper checks its own work -making sure that it has successfully navigated to the correct position in the tissue and that the images are of acceptable quality. Using this software, we have been able to collect image volumes from a wide range of ATUM-collected tissue libraries (including a mouse cortex UTSL, a mouse cerebellum UTSL, a mouse thalamus UTSL, and a larval zebrafish UTSL). These 3D image volumes ranged in size from about 1 to 100 terabytes of image data and required the imaging of many thousands of ultrathin sections.

## Sample preparation

### Tissue processing

All experiments were performed according to the guidelines of the Harvard Animal Care and Use Committee. The tissue samples for ATUM are standard EM blocks preserved using aldehydes, stained with osmium tetroxide, and embedded in a hard resin (Hayat, 2000). For large volumes to be imaged quickly, good contrast is essential. We often use a combination of the (R)OTO technique for enhancing osmium staining *en bloc* and lead citrate post section staining (Tapia et al., 2012). It is important to note, that by thickening and darkening membranes, this technique can make synapses more difficult to identify in single sections. A great deal of this ambiguity is resolved when a synapse is reconstructed in its 3D context. There are also a number of staining techniques that can be used to enhance synaptic labeling. Regrettably, most EM protocols were not developed to penetrate volumes that extend for 100 s of microns in depth. The lack of uniformity in staining, in general, can be a significant problem. More uniform staining can be achieved by decreasing stain concentration and increasing incubation time. However, with any particular sample there is a significant risk that the stain will not be acceptable all the way through the volume. One of the benefits of generating the low resolution image series with WaferMapper is that the thousands of sections can be evaluated for tissue quality before the more time-consuming high resolution imaging is begun. Once high resolution imaging is completed, WaferMapper can also be used to reimage ambiguous structures under different imaging conditions or after further post section staining.

### Sectioning and sample preparation

ATUM uses a reel-to-reel conveyor belt to collect sections from the water boat of standard ultramicrotome diamond knives. Once sections are cut and float into the water boat, they come in contact with the inclined surface of the moving collection tape that juts out of the water (Figure 1). Depending on the size of the tissue block, 1000–10,000 sections can be collected over a 24 h period with no human interaction. Once the ATUM sectioning and collection process is started, the operator typically leaves the room and can check in on cutting remotely via video. Knife water level is maintained automatically by a video feedback mechanism controlling a digital syringe pump. After sectioning about 100 μm of tissue, the microtome reaches the end of its useful range and has to be manually reset. At this time, the sample can also be moved to a fresh position on the diamond knife so that knife sharpness does not become a problem. In this way, large volumes can be sectioned with only a single interruption every several thousand sections. For some samples, it is possible to collect thousands of sections without tissue loss. However, many factors such as heterogenetity in the tissue, and knife dullness can result in sections breaking or folding as they are being cut. For most connectomics applications we are able to tolerate one or two damaged sections per hundred as long as damage is not occurring in sequential sections.

The tape containing the sections is next cut into strips and mounted on 100 mm diameter silicon wafers which are flat, conductive (doped) and vacuum safe. To adhere the tape to the wafer, the surface of the wafer is covered with double sided conductive tape. Each section needs a path to ground or it will become electrically charged during SEM imaging. This grounding can be accomplished by thin-film depositing a carbon coating over the entire surface of the wafer with tape strips attached (typically works well with backscattered electron detection). If the tissue will be imaged using voltages that cannot penetrate a carbon coating (see section: Imaging Hardware), use of a collection tape that is pre-coated with a conductive layer is required. The top surface of the conductive tape can then be connected to ground by using conductive tape or paint along its edges. Because this approach also works with backscattered electron detection, we are able to first use backscattered imaging to acquire large field of view overview images with minimal field distortion (because of high electron voltages) and then switch to secondary electron imaging if it is optimal for the smaller field of view high resolution imaging step.

Once the tape segments are mounted onto silicon wafers, we affix fiducial markers (Copper Reference Finder TEM grids style H6 from Ted Pella work well) to the double sided carbon tape at the corners of the wafers. This is critical for the wafer mapping process, described in detail below. A standard wafer box (Figure 1) can hold 25 silicon wafers containing hundreds of sections each, resulting in a 10,000-section UTSL that can be stored in a desk drawer.

## Imaging hardware

There are several SEM imaging systems commercially available that could be used for imaging ultrathin section libraries generated by ATUM sectioning. The software presented here was developed to drive off-the-shelf Sigma and Merlin SEMs (Carl Zeiss Microscopy LLC, Oberkochen, Germany) fitted with Fibics scan generators (Fibics Inc., Ottawa Ca.). All results presented here were imaged with one of these microscopes. The Fibics scan generator allowed for images to be acquired with pixel dimensions up to 32 by 32 k. Both of these microscopes have multiple detectors that include an outside-the-lens secondary electron detector, a below the lens backscatter detector, and an in-lens secondary electron detector. We use the in-lens secondary electron detector for high speed imaging because this detector's response speed was sufficient to keep up with high scan rates (10 MPS in the case of the Sigma and 20 MPS in the case of the Merlin). When imaged with this detector, our samples yielded the best signal using 1.7–3.5 keV and therefore required collection on an ATUM tape having conductive coating rather than thin-film carbon coating the sections after collection. We also found that the “depth of field” mode with extended depth of focus available with the Merlin is helpful for acquiring large fields of view of potentially uneven surfaces. Both microscope types were also fitted with an Evactron plasma generator (XEI Scientific, Redwood City, CA) to clean and etch surface material before imaging tape affixed to wafers.

## Imaging strategy and workflow

**Figure 2:**
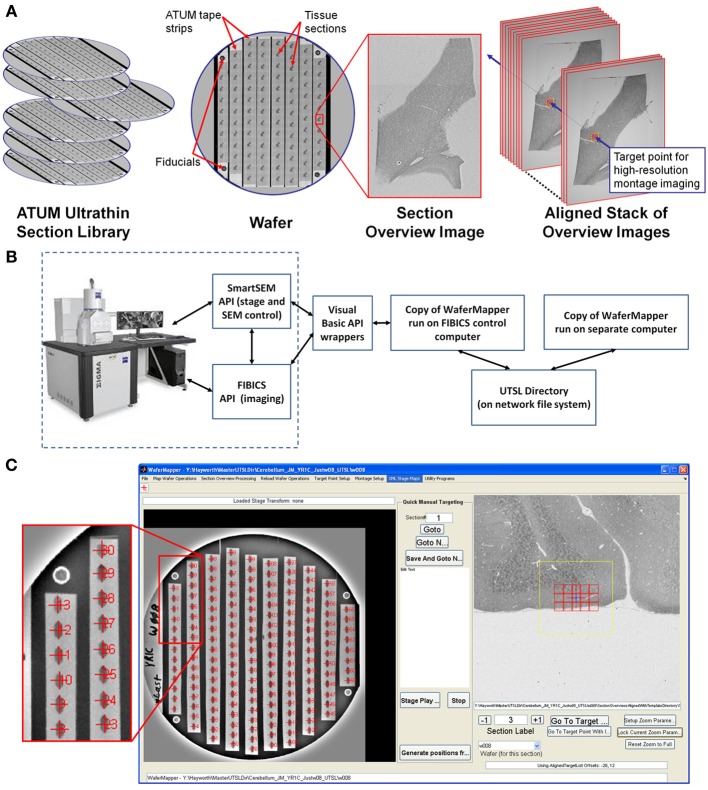
**Overview of WaferMapper terminology, SEM system integration, and Graphical User Interface (GUI). (A)** Graphical depiction of some of the key terms described in the text. **(B)** Block diagram showing how one copy of the WaferMapper program is used to automate SEM imaging while a separate copy is used to handle offline tasks such a browsing the aligned section overviews stack and graphically planning montage imaging volumes. A central UTSL Directory structure organizes all metadata and images related to the library. **(C)** The WaferMapper GUI is organized around a wafer-level overview display (left) and a section overview display (right). Each tissue section on the wafer-level overview display is marked by a red cross along with its section number. In the section overview display, a red cross designates the target point for high-resolution imaging. In this case the red cross is overlaid by a smaller blue cross designating where autofocuses should occur (which, in general, can be offset from the target point). The yellow box in the section overview display denotes the field-of-view used for local target point alignment (see section: Target point setup). The red boxes in the section overview display are the high resolution montage tile positions defined in the montage parameters (see section: Montage parameters).

A consequence of automated sectioning is that far more tissue can be cut than can currently be imaged at the highest EM resolution. Because this tissue is mounted on silicon wafers and imaged with an SEM, it is relatively easy to image, store, and reimage the tissue. It is possible, therefore, to treat ATUM cut samples as an UTSL to be sampled on demand. For some experiments, large target areas might be imaged once at high resolution. Other experiments might involve various small high resolution volumes being fit onto a low resolution map of the total tissue volume. The experimental flexibility gained by the UTSL depends critically on developing the means to allow easy navigation within the digital volume. This section (Imaging strategy and workflow) contains an outline of the intent of the low and high resolution imaging steps. The following section (Implementation: WaferMapper) contains a detailed description of the WaferMapper implementation. Graphical depictions of some of the key concepts (UTSL, wafer, fiducial, section overview image, aligned stack of overview images, target point) can be found in Figure 2A.

### Low resolution mapping

The first step in generating a UTSL is mapping the positions of each of the sections on all the silicon wafers. The low resolution mapping step can be accomplished using either an optical image or an EM image montage of the entire silicon wafer. Sections are identified automatically and then any instances of unlabeled sections or debris labeled as sections are corrected manually. This procedure marks the position of each section. For the microscope to consistently find the mapped section positions, reference points (fiducials) are imaged on each wafer (Figure 2A). Any time a mapped wafer is loaded into the SEM, the fiducial points are re-imaged and compared to the original fiducial images to determine the correction factor required to translate the wafer map coordinate system onto the new wafer position.

The second step in creating a UTSL requires obtaining a more detailed low resolution image of each section (but not the whole wafer). For this imaging phase, the microscope automatically uses the map of section positions obtained for each wafer (see above) to drive the microscope stage to each section on a wafer and obtain a “section overview” image (see Figure 2A). The overview image generally includes all of the tissue in the section (typically several square millimeters) while using a pixel size just small enough to identify features relevant to the future targeting of image volumes within the section (commonly 1 μm pixel size). After acquiring the overview images they are digitally registered to each other to remove effects of inter-section rotation and translation. The resultant “aligned stack of overview images” (see Figure 2A) constitutes a 3D coordinate map of tissue locations used for all subsequent navigation of the UTSL.

### High resolution image acquisition

With the low resolution UTSL overview map it becomes possible to select one or more target regions for higher resolution imaging throughout the volume. A center position “target point” (see Figure 2A) for a targeted region is recorded and in some cases a second higher resolution local registration (setting translation parameters only) is performed now with the target position at its focus. At high resolution, a target region may require imaging a mosaic of multiple overlapping images arranged in rows and columns. Imaging the target region therefore requires defining “montage parameters” (i.e., size of the imaging mosaic, resolution, and other imaging parameters). Once these parameters are set, each wafer can be loaded onto the microscope stage and imaged automatically.

High resolution data collection begins with loading a wafer and imaging the fiducials to adjust the wafer's section overview map to register it to the new position of the wafer on the microscope stage. These adjustments are typically in the range of 100 s of microns. With the montage parameters loaded, the microscope now has sufficient information to acquire a series at high resolution for each loaded wafer. Once the high resolution imaging step is initiated, the microscope automatically moves the stage to the first section and then moves to the target position. The scan rotation based on the stored parameters is initiated so that all the sections are acquired in the same orientation. The microscope then automatically adjusts focus and stigmation at the target region.

Correct focus and stigmation are critical for SEM imaging of tissue sections spread across ATUM tape strips and adhered to silicon wafers. The depth-of-field in typical SEM imaging is small relative to typical wafer and tape mounting variability. The beam focus must therefore be adjusted to match the z-position of the tissue as the stage moves across millimeters of tissue and centimeters of silicon wafer. Generating a high resolution beam spot also depends on stigmators compensating for any aberrations in the focusing of the electron beam. Our experience is that the optimal stigmation changes during an imaging period as the focus depth and microscope conditions change. Acquiring a dataset with consistent image quality therefore requires periodic automated focusing and stigmation.

We find that, when we apply the focus algorithms currently available on the Zeiss Sigma and Merlin, our focus quality is unacceptable about 5% of the time. Therefore, high resolution images undergo an automatic quality check which can trigger a corrective focus and stigmation followed by re-imaging. Because focus and stigmation can take as long as imaging time and failure rates can vary with tissue and imaging conditions, a variety of focusing strategies are made available (described below). When the high resolution imaging of a section is completed, the digital data is automatically moved from a local data buffer to long-term network storage.

If high resolution imaging is interrupted at any point, no data is lost. The wafer can be removed from the microscope for storage and then reloaded when convenient. Because this procedure exposes the wafer to air and can affect the microscope chamber and column conditions, there may be a delay of an hour or so before imaging conditions are ideal. We have been able to return to a wafer after years of bench-top storage for reimaging. However, it is likely that storing a UTSL for many years without degradation will require placing tissue sections in a desiccation or vacuum chamber. Tissue contrast can be altered by the initial imaging process (particularly at focus points), however this change is usually not destructive as long as surface contamination of the wafer is minimal.

## Implementation: WaferMapper

The software that oversees the steps outlined above is a MATLAB^®^-based program called WaferMapper. The MATLAB^®^ script allows researchers with limited programing background to readily customize the code according to their particular needs. The WaferMapper source code is freely accessible through a Google code SVN server (https://wafermapper.googlecode.com, See user guide for Matlab toolbox dependencies.) and we encourage any interested parties to participate in the further development of WaferMapper. A detailed, step-by-step user's manual is also available at this site.

In addition to the MATLAB^®^ code, we provide two C wrappers for interacting with the Zeiss SmartSEM API (to control the microscope) and the Fibics scan generator API (for acquiring high pixel density images). Although WaferMapper was written to drive the Zeiss/Fibics SEM system, it can be adapted to other imaging systems with the addition of appropriate command wrappers. For those who wish to build their own imaging software, the following description of our implementation should still be helpful as a practical guide to managing and imaging a UTSL.

### Overview

Figure 2B is a block diagram showing how WaferMapper interfaces with SEM hardware. WaferMapper can be run as a standalone application for steps which do not require SEM control. A UTSL directory structure, stored on a network file system, organizes all metadata and images related to a particular library. Figure 2C shows the WaferMapper graphical user interface (GUI). The GUI is organized around a wafer-level overview display and a section overview display. The red crosses designate the position of mapped sections. When WaferMapper is connected to the SEM and the currently loaded wafer is displayed, the user can quickly move the SEM stage position to any point on the wafer by clicking on the wafer image or to any point in a section by clicking on and zooming in on the section overview display. When WaferMapper is run on a computer not connected to the SEM, the user can browse through all wafer images and through the entire stack of aligned section overviews to graphically define a target region for high resolution montage imaging.

**Figure 3:**
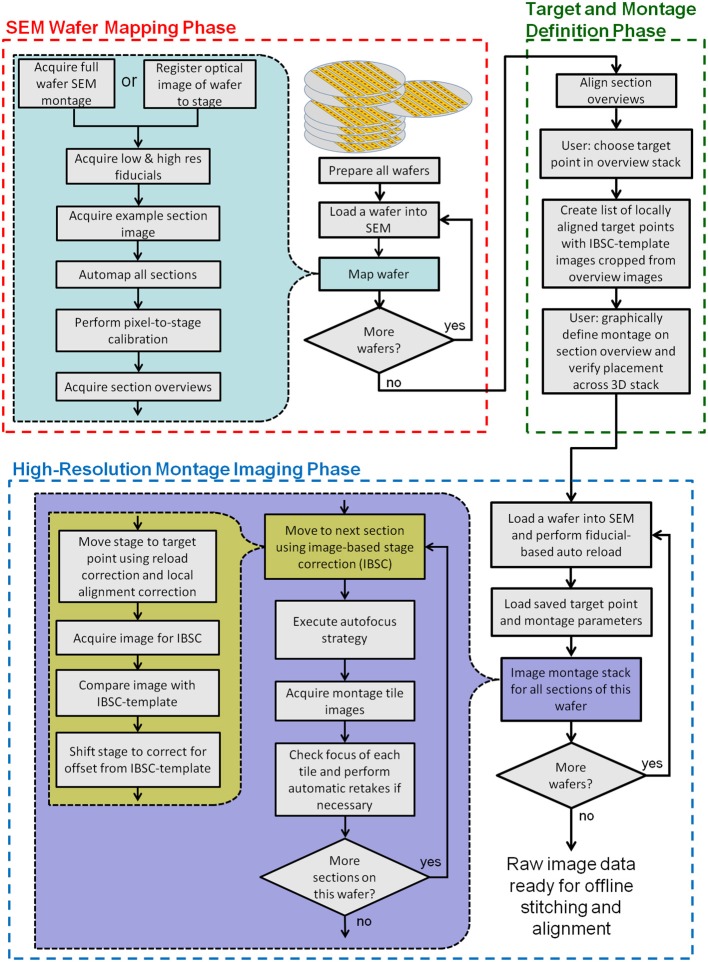
**Flow chart showing all key steps in the creation and imaging of a UTSL using WaferMapper**. The process is broken into three phases: an “SEM Wafer Mapping Phase,” a “Target and Montage Definition Phase,” and a “High-Resolution Montage Imaging Phase.” IBSC, Imaged Based Stage Correction (see section: Starting high resolution image acquisition).

Figure 3 is a flowchart showing all key steps in the creation and imaging of a UTSL using the WaferMapper software. Conceptually, the process is broken into three key phases. The first is an “SEM Wafer Mapping Phase” in which the software is used to map out the locations of all sections across all wafers in the library, and in which low resolution overview images of all sections are acquired by automation of SEM stage movements and imaging. The second is a “Target and Montage Definition Phase” in which WaferMapper (usually being run on a non-acquisition computer) is used to align all section overviews and is used to graphically define a subregion for high resolution montage imaging. The final phase is the “High Resolution Montage Imaging Phase” in which WaferMapper automates the SEM operations necessary to acquire the defined high resolution volume data. Each of these phases is described in detail below.

### SEM wafer mapping phase

The goal of the “SEM Wafer Mapping Phase” is to produce a set of images and metadata covering all wafers and all sections in a UTSL. This collection of images and metadata (e.g., stage coordinates of all section overview images and fiducial images, pixel-to-stage calibration scaling factor, pixel size, dwell time, etc.) allows reloading of wafers and automatic movements of the stage to preselected target points within each section. Because a UTSL may consist of tens or hundreds of wafers and many thousands of sections, we have tried to automate the majority of the steps in this process.

#### Acquiring a full wafer image

**Figure 4:**
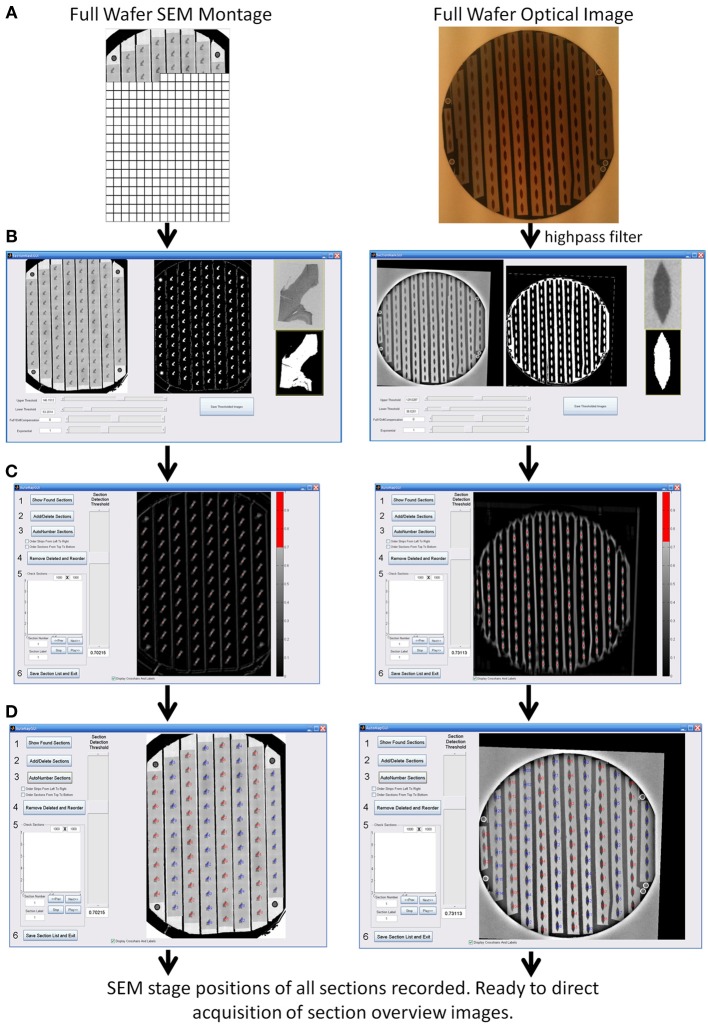
**WaferMapper process steps for automatically finding the positions of all sections on a wafer. (A)** Acquisition and registration of a full wafer image to the SEM stage using either a full wafer SEM montage (left) or a full wafer unprocessed optical image (right). **(B)** Thresholding of full wafer image and example section image creating binary masks. **(C)** Convolution of the example section mask over the full wafer image creates a heat map whose hottest points correspond to likely section locations. **(D)** After the user selects a suitable threshold for the heat map, the program marks the locations of all centroids as individual sections and numbers the sections in strips according to the order the tape strips were collected on the ATUM machine.

An image of the entire surface of the wafer is acquired before mapping of sections begins. This image can be generated optically or using an electron microscope. The benefit of generating the image in the electron microscope is that contrast will be based on electron scattering, i.e., metalized tissue will stand out from the background. However, full wafer imaging is time-consuming (~20 min on the Merlin and 60 min on the Sigma) given that the limited field of view of an electron microscope necessitates acquiring many individual montage tiles. We have often found, however, that even images of the wafer rapidly taken with an optical camera while the wafer is being lit indirectly via a diffuse white background are of sufficient quality to serve as a full wafer image so long as all of the ultrathin sections and fiducials are visible in the image (Figure 4A). The regularity and resolution of the full wafer image will determine how accurately the next stage of mapping, overview image acquisition, can be targeted. We typically acquire overview images with a field of view approximately a millimeter larger than the tissue section which corresponds to about a half millimeter of fault tolerance in the full wafer image.

If WaferMapper already has access to an optical image of a loaded wafer, the user can navigate to “Map Wafer Operations” > “Acquire Full Wafer Montage” and select the option to load that camera-acquired full wafer image. The full wafer image must then be mapped into the microscope's stage space by selecting three or four fiducial points on the wafer's image and manually driving the stage to the corresponding locations. To create a new wafer image within the SEM, the user can first select “Map Wafer Operations” > “Wafer Parameters” to define the image resolution and dwell time of the full wafer image. The user then navigates to “Map Wafer Operations” > “Acquire Full Wafer Montage,” defines the edges of the wafer image and begins montaged acquisition.

Once a full wafer image has been mapped onto stage space or acquired within the SEM, the wafer image can be used for navigation. The user can select “Map Wafer Operations” > “Free View” and then click on any point in the wafer image to drive the stage to the corresponding location.

#### Acquire images of wafer fiducial marks

At the core of a mapped UTSL is a set of related coordinate systems which allows every point in the aligned stack of section overview images to be mapped back to a particular position of the SEM stage. Since there is typically a significant offset introduced when reloading a wafer into the SEM, we also acquire a set of images of fiducial marks that are permanently placed on the corners of the wafer. The stage positions of these fiducial points are used as a reference frame for all other stage positions. The first step of acquiring overview images is, therefore, to image the fiducial points on the wafer first at low resolution (3 μm per pixel), then at high resolution (0.25 μm per pixel). By selecting “Map Wafer Operations” > “Acquire Low Res. Fiducials,” the user can use the full wafer image to navigate to fiducial points on the wafer and acquire images of these points. These images can then be used any time the wafer is reloaded to map section overview space back onto microscope stage space.

#### Automap all sections

Once the fiducials have been imaged, the next step is to automatically determine the positions of all sections on the wafer. The user selects “Map Wafer Operations” > “Acquire Example Section Image” and either is directed to cut out an example image of a section from the full wafer image (if a full wafer optical image is being used) or is able to acquire a new example image of a section by SEM (if a full wafer SEM montage is being used). This example section image is then used as a template which is scanned across the full wafer image at a range of image rotations to pick out the positions of the other sections with sufficient precision to drive section overview imaging. This process is displayed in Figure 4 for an example wafer whose whole wafer image was obtained by a full wafer SEM montage (left), and for an example wafer whose whole wafer image was obtained by an optical camera while the wafer was lit indirectly via a diffuse white background (right).

The “Map Wafer Operations” > “Threshold image” command calls up a new GUI (see Figure 4B) which is used to set upper and lower gray scale thresholds to convert both the full wafer image and the example section image into binary masks. Next the user selects “Map Wafer Operations” > “Auto Map All Sections” to open up the automap GUI (see Figure 4C). Within this GUI, the example section binary mask is used as a convolution kernel and convolved at multiple rotations across the full wafer binary mask. The result is a heat map image whose “brightest” points correspond to high correlations between the wafer image and the example section image. Bright points in the heat map image correspond to locations on the wafer image that resemble the example section. The user selects a threshold for heat map image and the centroids of image patches that pass threshold constitute the section locations (see Figure 4D). With mouse clicks and zoom operations, the user can add or remove section positions to correct any mistakes made by the automatic section finding. On most wafers there will be a few additions and subtractions to the section list. Once all of the sections are marked, the sections are automatically assigned number labels according to their position on the collection tape. Typically this automap process will find and correctly label >95% of sections on a wafer. Sections that are not identified automatically, usually because they lay close to a high contrast edge or because two sections are too close together, can be identified manually and added by the user with a few mouse clicks.

#### Pixel-to-stage calibration

To use overview images of sections to direct stage positioning, the relationship between pixel size and stage travel must be precisely defined. Slight inaccuracies, arising from imperfect calibration of the microscope, can result in noticeable errors in WaferMapper's ability to target the correct region of tissue. To compensate for potential discrepancies between pixel size and stage travel, we perform a pixel-to-stage calibration immediately before the acquisition of the section overview images. This process produces a pixel-to-stage conversion factor which is specific to the particular set of imaging conditions used for the overview images. The pixel to stage conversion factor is determined by selecting “Map Wafer Operations” > “Perform Pixel to Stage Calibration.” The user is then prompted to select an image target. The microscope takes an image of the target region using the same settings that will be used to acquire the section overview images, moves the stage a defined distance and then takes a new image. By comparing the displacement in the images, a pixel-to-stage conversion factor is obtained and recorded as part of the metadata associated with this wafer.

#### Acquire section overviews

With fiducials mapped, sections identified, and the stage calibrated, WaferMapper has all the information it requires to begin acquiring an overview image of every section on the wafer. When the user selects “Map Wafer Operations” > “Acquire Section Overview Images,” WaferMapper drives the stage to the first section and begins acquiring images using the user-defined settings for this UTSL. The default setting is to acquire 3 mm-wide images with pixel sizes slightly smaller than 1 μm. These settings can be changed according to the size of the target sample, the accuracy of the section targeting and the desired precision of the 3D map of the UTSL. Depending on the number of sections and the imaging parameters, the process of acquiring section overviews typically takes 30 min–3 h per wafer depending on the number of sections and the desired overview image quality.

#### Repeat for all wafers

**Figure 5:**
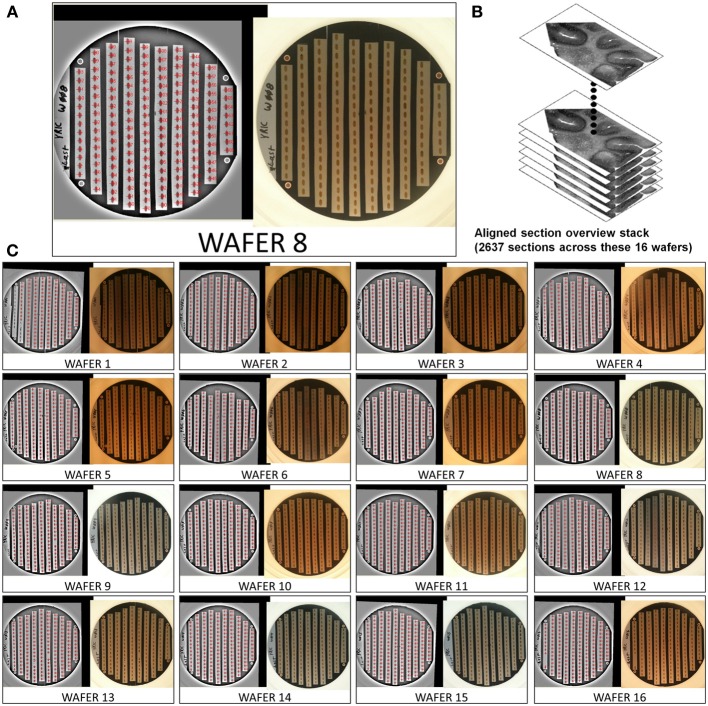
**Example of a 2637 section UTSL of mouse cerebellum tissue spanning 16 wafers**. An optical image of each wafer was taken and used as the basis of automapping all sections. **(A)** Optical image of wafer 8 (right), filtered optical image with automapped positions of all sections labeled (left). **(B)** Graphical depiction representing the stack of 2637 overview section images acquired during the mapping phase. **(C)** All 16 automapped wafers in this UTSL.

**Figure 6:**
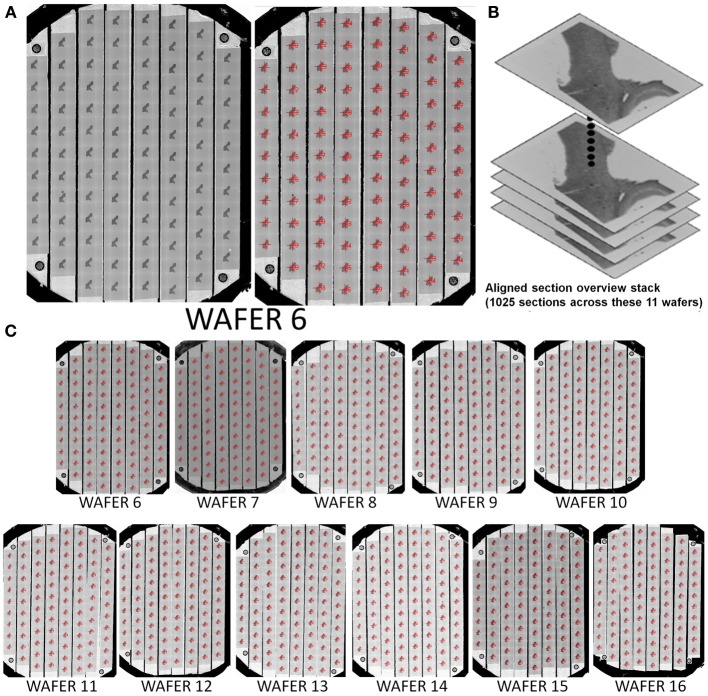
**Example of a 1025 section UTSL of mouse cortex tissue spanning 11 wafers**. A full wafer SEM montage image of each wafer was taken and used as the basis of automapping all sections. **(A)** Full wafer SEM montage of wafer 6 (right), same image with automapped positions of all sections labeled (left). **(B)** Graphical depiction representing the stack of 1025 overview section images acquired during the mapping phase. **(C)** All 11 automapped wafers in this UTSL.

This mapping procedure is repeated for every wafer in the UTSL. Figure 5 shows an example of a fully mapped UTSL consisting of 2637 sections of mouse cerebellum tissue spanning 16 wafers. The mapping procedure for this UTSL was based on optical camera images of each wafer. Figure 6 shows an example of a fully mapped UTSL consisting of 1025 sections of mouse cortex tissue spanning 11 wafers. The mapping procedure for this UTSL was based on full wafer SEM montage images of each wafer.

### Offline target and montage definition phase

With the mapping data acquired, the section overview images can now be assembled into a 3D map of the tissue volume in which high resolution imaging targets can be selected.

#### Align section overviews

Each section overview image must be aligned to its neighbors across all wafers in order for the stack of images to be treated as an image volume. Here we describe the cross-correlation based alignment strategy that we have used to acquire all data sets to date. For this method, we find the y translation, x translation and rotation that produces the highest cross-correlation value between two images.

Typically, the section overviews for a particular wafer are aligned on a non-acquisition computer with access to the UTSL directory while other wafers are being imaged. To align images, the user first selects a template image either from the current wafer (if this is the first wafer mapped in the UTSL) or from the previous wafer which has already undergone section overview alignment. By aligning each wafer to a section in the previous wafer, a single aligned stack is created spanning all wafers in the UTSL.

At this stage in alignment, each section on the wafer is aligned to the selected template image. The section-to-section registration produced by this alignment is not as good as an alignment procedure that compares neighboring sections, but the template matching has the advantage of not accumulating drift and being robust to single “problem” sections in the stack. The automated alignment of each wafer usually takes 10–30 min using 3.2 GHz processors on a standard desktop computer.

Any mistakes in the alignment can be corrected using “Section Overview Processing” > “Check and Correct Alignment GUI,” which calls up an easy to use GUI in which alignments can be quickly reviewed and corrected by simple mouse drags. It is not necessary, at this point, that the resulting alignment is perfect. The alignment only needs to be good enough that, when an imaging target point is chosen on one section overview, a second stage of target point alignment (described below) can access the appropriate region of each section overview.

By following the above procedure, a small UTSL can be mapped in several hours. A larger dataset, consisting of ~10,000 sections might take closer to 1 weeks (working 8 h per day). The end product of the mapping process is a set of full wafer images that can be used to navigate around each wafer and a low resolution 3D image volume of the tissue sections that can be used to direct high resolution image capture.

#### Target point setup

The first step of defining a target point for high resolution imaging is to choose an XY position from within a section overview image. This XY position, the “target point,” will be used as the reference point for targeting high resolution imaging and will serve as the center of a second stage of more precise local alignment of the section overviews. The user selects a target point by loading a UTSL and wafer into WaferMapper and selecting “Target Point Setup” > “Choose Target Point in Aligned Section Overview.” The user is then prompted to click on a point within the displayed section overview image and can save the target point for later use. Unlike the previous steps in the wafer mapping process, in which it is expected that a single map is generated for a given UTSL, the selection of target points is a branch point where many target points can be defined, one for each high resolution subvolume to be imaged.

Once a target point is selected, a new targeted alignment is executed by selecting “Target Point Setup” > “Generate and Save List of Aligned Target Points.” For this alignment, a relatively small window is extracted from each section overview image. Each subregion of the section overview images is aligned to a running average of previously aligned subregions. This process takes about 10 min per wafer. The goal of this second stage of alignment is to produce a better local section to section registration than can be generated from an alignment of the entire section overview images themselves. Once this alignment is completed, any mistakes can be corrected using the “Target Point Setup” > “Check and Correct Target Point Alignment” GUI.

The results of each target point alignment are stored in a new Aligned Target List subdirectory in the UTSL containing all of the aligned subregion images for use in image-based stage correction (IBSC), described below, and a new datafile called “AlignedTargetList.mat.” “AlignedTargetList.mat” contains the pixel offsets needed to align each of these cropped subregions. These pixel offsets (when combined with the pixel-to-stage calibration factor determined during the mapping phase) will be used in the high resolution imaging phase to quickly position the SEM stage for imaging each section.

#### Montage parameters

**Figure 7:**
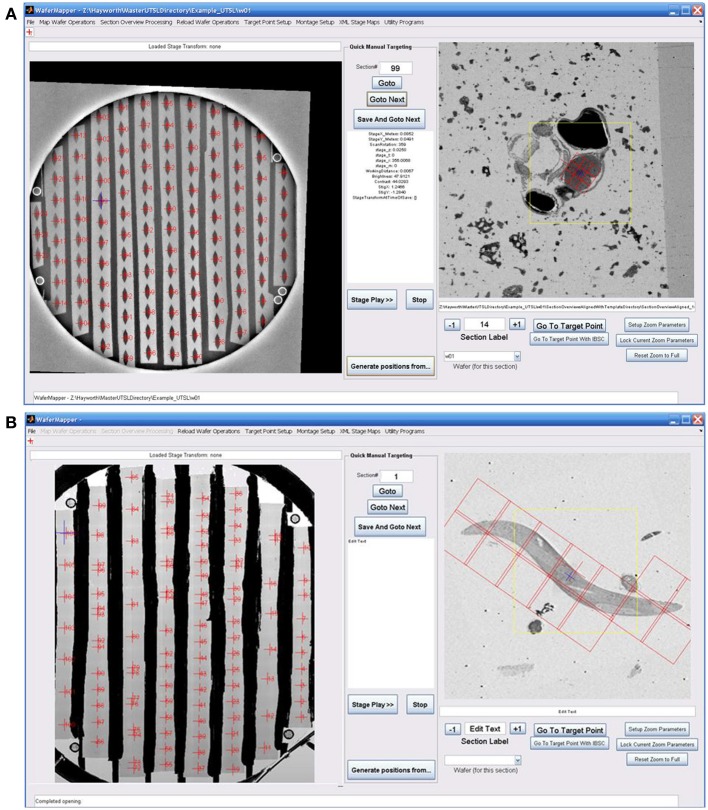
**Examples of graphically defining an imaging target and montage. (A)** Image shows the WaferMapper GUI being used to graphically define a 3 × 3-tile montage (red boxes) covering just the brain region in a larval zebrafish UTSL. **(B)** Image shows WaferMapper used to graphically define a 3 × 7-tile montage (red boxes) covering a single worm in a *C. elegans* UTSL. Note the ability to selectively designate a subset of montage tiles for imaging. Of crucial importance is the software's ability allowing the user to scroll through the entire stack of section overviews to graphically check placement of the imaging montage across all sections prior to the start of any long imaging run. The yellow box in the section overview display denotes the field-of-view used for local target point alignment (see section: Target point setup). The small blue cross denotes the autofocus position to be used, which in this case is deliberately offset from the center of the montage.

The position, dimensions and imaging conditions of each high resolution dataset are defined within “Montage Setup” > “Set Montage Parameters.” The central position of each image montage is set relative to the aligned target point by defining an X offset, Y offset and North Angle. The dimensions of the montage are set by defining the field of view of each tile (“Tile FOV”) and the number of rows and columns of tiles in each montage. Additionally, the overlap between tiles is defined here. For our systems, four micrometers of overlap was sufficient to consistently acquire images without gaps between tiles. WaferMapper provides a graphical overlay of the montage tile positions on top of its section overview display (Figure 7), allowing the user to scroll through the entire stack of section overviews and graphically check placement of the montage tiles across all sections prior to the start of a long imaging run.

The pixel size (“Tile FOV”/“Tile width”) and pixel dwell time must be set to achieve the necessary balance between image scanning time and image quality. Using high contrast staining and the in-lens secondary electron detector we were able to obtain images with acceptable noise levels using the maximum scan speed of our microscopes (50–100 ns), however these results depend heavily on the tissue preparation and imaging configuration. The Fibics scan generator allows image sizes up to 32 × 32 k pixels, which means 100 μm-wide images can be acquired at 4 nm per pixel resolution. Being able to scan large field-of-view images at high resolution reduces the impact of tile to tile overhead on image acquisition time and generally increases the efficiency of managing large datasets.

WaferMapper includes the option to take an overview image of the targeted imaging region before the high resolution imaging begins. Being able to take advantage of this option requires that the microscope setup that is used to acquire high resolution images is also amenable to large field of view imaging.

### High resolution montage imaging phase

#### Reload

Once the section overviews have been acquired, the wafer can be removed from the microscope and stored. When the wafer is placed in a new SEM or returned to the same one, the position of the wafer on the stage will not be exactly the same as when the wafer was mapped. To bring the wafer map into register with the new position on the stage, the user can follow the steps listed under the “Reload Wafer Operations” menu. The first step is to manually set the coarse offset of the stage. The microscope drives to the first fiducial point on the wafer and the user manually rotates and translates the stage to correct for any gross change in position. “Free view with Offset” can then be used to confirm that the new wafer position is roughly correct. Once the stage rotation and translation have been adjusted within approximately a millimeter of the mapped position, the user can then run “Do all steps for Stage Correction.” The microscope will then automatically drive to the fiducial positions, take new images and compare these images with the original images of the fiducials. This comparison is used to find a coordinate transformation that will be used to translate between stage space and wafer map space for the remainder of the imaging session. This ability to automatically register a reloaded wafer is also crucial when it is necessary to retake images, when imaging a new region, and when sharing a UTSL between different laboratories. Once reloaded, a wafer's metadata, in principle, contains all the information necessary for another lab with the same SEM setup to replicate an imaging run.

#### Quality check

The ability to acquire a high quality large-scale SEM dataset depends heavily on imaging with the correct focus and stigmation settings. The depth of field of the SEM is typically around ~0.5–10 μm, depending on the imaging modality, so that multiple focus points might be required to image a large montage. Depending on the sample, imaging conditions and stability of the microscope, periodic refocusing and restigmating might be required regardless of the sample flatness. To acquire images for days without human intervention, WaferMapper uses a variety of strategies to minimize blurry images.

**Figure 8:**
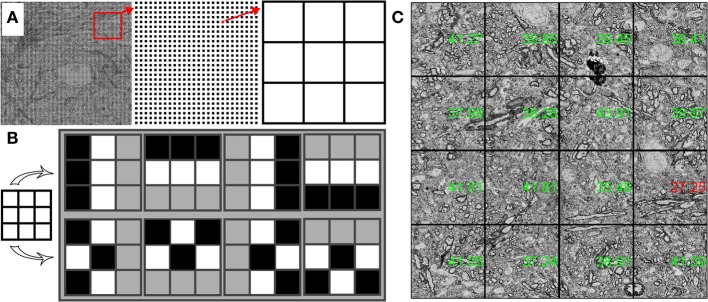
**Structure of quality check procedure. (A)** A 200 × 200 array of 3 × 3 pixel kernels is extracted from an SEM image. **(B)** Structured (top) and unstructured (bottom) contrast patterns are compared to obtain a quality value for each 3 × 3 pixel kernel. **(C)** Stage stitched montage of images displayed with quality values in green (passing) and red (below threshold).

WaferMapper includes an algorithm that evaluates the quality of SEM images. We designed this algorithm to be able to quickly judge the quality of very large images and then to base its evaluation not on the average quality of the image, but on the quality of the best regions of the image. By only paying attention to areas with the most high frequency contrast, quality values are less sensitive to changes in tissue statistics that might come from blood vessels or section edges. For the quality check to read and analyze images without adding to the tile-to-tile overhead time, the quality check samples only a small fraction of the available pixels. Quality check reads in a grid, usually 200 × 200, of 3 × 3 pixel image kernels from a newly acquired image (Figure 8A). These samples are then fit into a 200 × 200 by 9 image volume.

To find a quality value for each grid point, we find the relative contrast of different patterns within the 3 × 3 pixel kernel. One set of patterns finds the difference in the average intensities of adjacent lines of pixels (two horizontal comparisons and two vertical comparisons). The other set of patterns compares the difference in the average intensities between groups of interleaved pixels (Figure 8B). A well-focused SEM image of cell membranes will tend to produce relatively more contrast in the adjacent lines pixel patterns than in the interleaved pixel patterns. Once a quality value is obtained for each grid point, a quality value for the entire image is obtained by averaging a small percentage of the best grid points. In this way, changes in tissue statistics have a relatively small effect on the quality rating. This method can also be used to identify poor quality regions of the tissue or of the imaging field.

Within WaferMapper's “Montage Parameters” setup menu, the user can choose to have the quality check performed at several points in the image acquisition process. First, a quality check can be performed after each autofocus to determine if the correct working distance has been obtained. After each autofocus, WaferMapper can take a quick image and perform a quality check. If the quality value exceeds the user-defined threshold, high resolution imaging begins. Otherwise, the image is refocused. Second, the user can choose to have a quality check performed after each tile is acquired. If the image quality fails to pass a user defined threshold, the image is refocused and retaken. Finally, the quality values of each tile are displayed on the down-sampled stage-stitched image of the montage (Figure 8C). Stage stitched images, with the quality values displayed on top, provide an easy way for the user to monitor the imaging process and to review the performance of the microscope.

#### Focusing strategies

The best possible image quality will usually be achieved by autofocusing, then autostigmating and then autofocusing again (focus-stig-focus) before each tile is acquired. However, this procedure can take significantly longer than the acquisition of a single tile. Therefore, a variety of strategies are offered in WaferMapper for increasing the efficiency of high throughput data collection. In many cases a single autofocus before each montage will be sufficient to produce acceptable image quality. For large montages a three-by-three grid of focus points can be acquired and then fit to a plane that predicts the optimal focus point for each tile of the large montage. Alternatively, tiles can be pooled and a central focus point acquired before a two-by-two box of tiles is acquired.

In a typical example we might choose to focus-stig-focus once per section as long as image quality stays above threshold. When the microscope starts a new section it will first drive to a pre-defined central focus point within the montage. The microscope then takes a quick image approximately the size of an image tile and drives to the region within the image with the highest contrast. In this way, WaferMapper avoids autofocusing on regions, such as the interior of blood vessels, which provide no useful information to the focus algorithm. The microscope then performs a focus-stig-focus and begins imaging the first section. The order in which the tiles are imaged is determined by the proximity of the focus point so that, if refocusing is required, best advantage is taken of each focus point. Once an image is acquired, the quality is evaluated. If the tile passes, the microscope moves onto the next tile. If the tile fails, the microscope refocuses and takes the image again. At any point in the imaging process a user can review either the images or the quality values being produced by the microscope and assign sections to be retaken. This strategy of only focusing once if the quality values stay above threshold works well when image acquisition time is small relative to the time it takes to autofocus and when the majority of tiles can be imaged using a single focus point.

#### Starting high resolution image acquisition

With the aligned target points loaded and the montage parameters set, WaferMapper is ready to acquire images. The user selects “Montage Setup” > “Acquire Montage Stack Main” and is prompted to select a target directory. This target directory can be anywhere; however, image writing and quality check work best if the data is saved on a local solid state drive. This data can then be managed and transferred through a network connection to a large data server. In addition to writing the images in this directory, a log file is written that records all stage movements, image qualities and image conditions.

When starting image acquisition, the user will also be asked whether or not WaferMapper should use IBSC. The accuracy of targeting we were able to achieve using wafer fiducials only was usually limited to about ±15 μm. We found that we could significantly improve this accuracy using IBSC. When this option is selected, WaferMaper acquires a quick image every time it drives to a new section. This image is processed using a difference of Gaussians filter to enhance features on the scale of cell bodies. This section's aligned subregion target image (cutout of the section overview image after local alignment during the Target Point Setup step, see section: Target point setup) is likewise filtered and compared with the newly acquired image using cross correlation. If the stage movement was completely accurate, then the two images should match exactly. If they do not, then WaferMapper uses the offset obtained from the cross-correlation to adjust the stage position and then checks its work with a second image. The accuracy with which this second image matches the section overview target is also recorded within the log file.

## Example data

**Figure 9:**
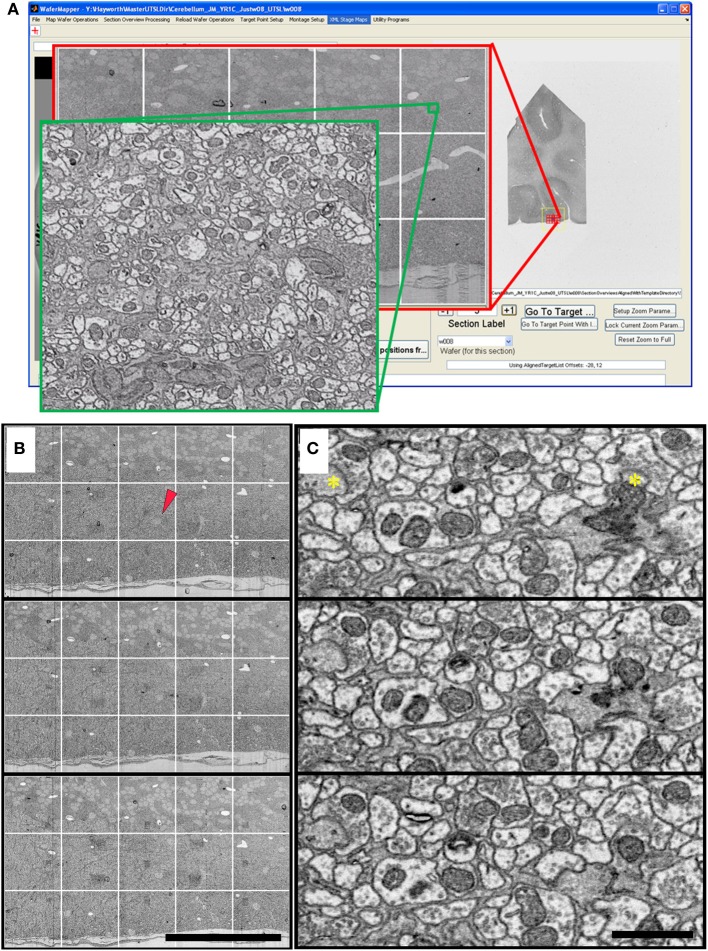
**Cerebellum UTSL example data. (A)** Image of WaferMapper GUI showing a graphically-defined 3 × 5-tile montage targeted near the surface of a cerebellar folium. Zoom overlays show high resolution images of that region. **(B)** Montage images of three successive ultrathin sections in this cerebellum UTSL acquired automatically by WaferMapper (scale bar = 100 μm). Red arrow shows location of corresponding high resolution images shown in **(C)**. **(C)** Cut outs of high resolution data imaged at 10 MPS and aligned in FIJI (scale bar = 1 μm). Asterisks mark position of two synapses.

**Figure 10:**
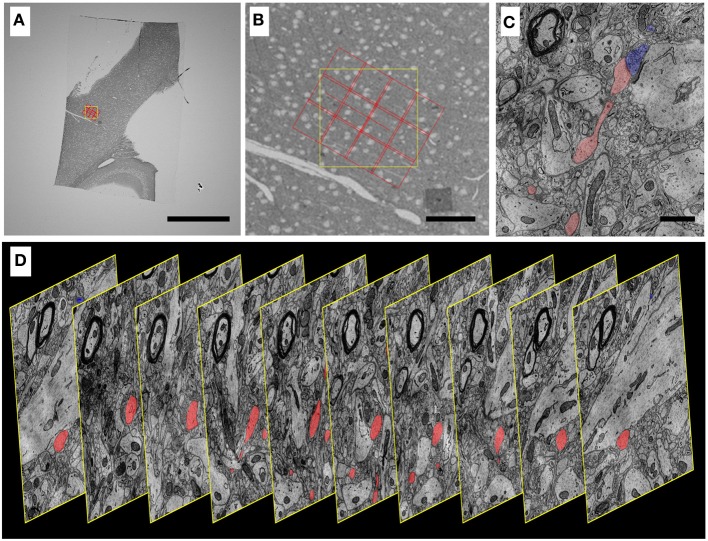
**Progressively higher resolution images through a mouse cortex UTSL mapped and imaged by the WaferMapper software. (A)** Single section of the 1025 section mouse cortex UTSL whose wafers are displayed in full in Figure 6. This image is from a screen capture of the WaferMapper program showing the location of a 3 × 4-tile montage overlaying a target region. (scale bar = 1 mm). **(B)** Zooming in on the section overview display in WaferMapper allows the graphical display of the montage to be finely positioned relative to blood vessel and cell body landmarks visible at the resolution of the section overview images. (scale bar = 100 μm). **(C)** Zooming in again, this time to a small region of one image from a larger stack of images automatically acquired by WaferMapper of this same UTSL. The outlines of two neuronal processes sharing a synapse are shown highlighted in color. (scale bar = 1 μ). **(D)** Graphic displaying every 10th image from this same aligned dataset acquired by WaferMapper. ATUM sections were cut at 30 nm thickness, thus these images are displayed here at 300 nm intervals through the tissue.

Data sets have been acquired using WaferMapper that range in size from 1 to 100 terabytes. Figures 9, 10 show some example images from WaferMapper runs of a mouse cerebellum UTSL and a mouse cortex UTSL. These figures illustrate the range of scales which must be spanned by the mapping software in order to precisely target automated imaging of a small montage volume within the much larger volume of the full UTSL. Below we describe the mapping, imaging and data set acquired from one test of WaferMapper on the mouse cerebellum UTSL shown in Figure 5. Table 1 provides actual average data acquisition times and rates for the various steps in the mapping and imaging process. These times were recorded during a separate, multi-month imaging project using the WaferMapper software.

**Table 1:** **Breakdown of the time required for each step of data acquisition using ATUM-SEM and WaferMapper**.

**Sample preparation**	**Time (varies with tissue size and staining method)**
Tissue processing	1–2 weeks
Sectioning	8–30 s/section
Constructing wafers	~30 min/wafer (100–500 sections per wafer)
**Wafer mapping**	**Time per wafer**
Full wafer image (optical)	3 min/wafer
Wafer loading into chamber	5 or ~45 min/wafer (with or without load lock)
Full wafer image (EM alternative to optical)	20, 60 min/wafer (Merlin, Sigma)
Section mapping	~10 min/wafer
Section overview image acquisition	30–120 min/wafer (depending on desired image quality)
Offline wafer mapping^*^	**Time per wafer (using 2 Intel Xeon x5672 3.2 GHz 4 core processors)**
Section overview alignment (automatic)^*^	10–30 min/wafer
Manual correction of overview alignment	0–20 min/wafer
Target point alignment^*^	10–30 min/wafer
Manual correction of target point alignment	0–20 min/wafer
**High resolution imaging**	**Time per wafer**
Wafer load into microscope	5 or ~45 min/wafer (with or without load lock)
WaferMapper reload procedure	~10 min/wafer
Montage setup	~5 min/wafer
**Montage time**	
Movement to section	~10 s
Image based stage correction	~1 min
Focus-stigmation-focus^*^	~2 min
Pixel dwell time	50–3000 ns (depending on tissue signal and detector)
Time between tiles (with quality check)	8 s
**Estimated project breakdown**	**High speed imaging of 10,000 sections using 100,000 × 100,000 pixel montages**
Raw pixel scan time (minimum)	57 days
Montage acquisition time (with overhead)	104 days
Total data acquisition time (start to finish)	130 days

*Asterisks indicate steps that could significantly benefit from further software development*.

### Cerebellum

The cerebellum of an adult mouse was cut into several 300 μm-thick vibratome sections and stained with osmium tetroxide and uranyl acetate. The tissue was embedded in Embed 812 (Electron Microscopy Sciences). A block containing one of these vibratome sections was trimmed to include a 1 × 3 mm wide region of cerebellum. The blockface was trimmed so that the leading and trailing edges of each section come to a point. This shape helps minimize cutting disruptions caused by the contact of the block face with the diamond knife and the removal of the section from the edge of the diamond knife. Eight thousand ultrathin sections were cut at a thickness of 30 nm and collected on carbon coated Kapton tape. A subset of this ATUM run's tape containing 2637 sections was cut into strips and mounted on 16 wafers as shown in Figure 5.

#### Mapping

The WaferMapper software was used to map these 16 wafers and acquire overview images of each section to generate a low resolution 3D map of the tissue. Viewing the aligned overview stack in WaferMapper, an imaging target point was selected from a region of the molecular layer of the cerebellum where the arbors of the Purkinje cells were parallel to the plane of microtome sectioning. By selecting “Target Point Setup” > “Generate and Save List of Aligned Target Points,” the software was used to generate a second, local alignment, suitable for directing the high resolution imaging.

#### Imaging

Using WaferMapper's GUI display of the aligned overview stack, we graphically defined a high resolution imaging montage that encompassed the arbors of several Purkinje cells (Figure 9A). The montage consisted of three rows and five columns of tiles. Each title consisted of 12,800 × 12,800 pixels resulting in a total 4 nm resolution montage that covered about 250 × 150 μm of cerebellum. High resolution images were collected from 498 sections spanning three wafers and ~15 μm of cerebellum (Figures 9B,C). The acquisition of the high resolution data required ~100 h of microscope time on a Zeiss Sigma. Approximately one third of this acquisition time was consumed by scanning voxels at 10 MPS. The remaining acquisition time was spent primarily on autofocusing. We found that this imaging time could be significantly reduced on a Zeiss Merlin due to its faster scan speed and larger depth-of-field.

## Future directions

### Software development

We wrote WaferMapper in MATLAB^®^ so that researchers who are not primarily programmers could readily add to the code according to the needs of their experiments. To date, each large dataset acquired with WaferMapper has involved modifications of the code. While the core version of WaferMapper we have released should be able to acquire most types of data, we see collaborative development of the code as critical to WaferMapper's usefulness as new technologies and new uses for ultrathin section libraries evolve.

WaferMapper meets our initial simple goal of producing a data acquisition pipeline in which more time is spent acquiring image montages than is spent finding the right place to image. However, even during the production of this software, there have been significant improvements in SEM scan speed and more improvements are on the way. The future development of WaferMapper will hopefully produce streamlined versions of the code as well as a version with fewer Matlab toolbox dependencies. At the time of the submission of this publication, the most pressing areas for further code development are finding faster and more reliable methods of both focusing the microscope, judging image quality, and aligning section overviews and target points (Table 1). A branch of the code being developed, primarily by Forrest Coleman, aligns SURF points over a large number of sections as an alternative to cross correlation to improve the targeting of high resolution imaging within UTSLs. We find that this solution is often more robust than the current implementation of cross correlation. This branch of the code and others will be made available through the Google Code SVN server. We encourage other users of the WaferMapper software to test code and to add to the repository as new solutions are developed.

### Stitching and alignment

Small EM volumes (<1 terabyte) can be aligned on a powerful desktop computer using publicly available alignment software such as the registration plugins for Fiji (Schindelin et al., 2012). However, the stitching and alignment of high resolution images becomes increasingly difficult as data sets become larger. The computational power required to manipulate and process terabytes of images requires hardware that is not standard in most labs and, while most steps in alignment are amenable to parallelization, running these steps in parallel often requires changes in code and expertise in managing clusters. Because of these problems, aligning multi-terabyte datasets is currently being done by only a few groups. However, the recent production of many multi-terabyte EM volumes has spurred efforts to scale up alignment tools to make it easier for the broader research community to turn hundreds of terabytes of EM images into usable 3D tissue maps.

### The promise of ultrathin section libraries for collaborative connectomics

A major goal of the integration of automated tape collection and automated imaging of sections is to make volume EM easy enough to be a standard technique that many labs can use to study biological samples that are tens or hundreds of micrometers wide. However, we also wish to stress the potential of UTSLs to allow a new type of collaborative neuroscience. As discussed above, the ATUM in a few days of cutting can potentially produce so many ultrathin sections that it might take decades to image them in total at high resolution. A typical research publication using ATUM-SEM and WaferMapper might end up acquiring high resolution images from only 1% of the total volume of a collected UTSL. For example, in the visual thalamocortical slice case outlined in the introduction one researcher may end up imaging and sparsely tracing only a finely targeted 300 × 300 × 300 μm volume in cortical layer IV -mapping out the local connectivity of thalamic afferents and interneurons in that area. Once this research is published, other labs may wish to build upon this connectomics data by performing additional imaging and tracing in neighboring regions of the same brain, literally starting their tracing work from the very same neurons in this already published study. In this way, a collaboration of multiple research labs could muster the time and resources necessary to elaborate the connectomes of larger inter-regional circuits than any one lab could by working alone.

In this paradigm, some research labs might specialize in producing UTSLs with the highest quality ultrastructure preservation and staining, encompassing brain regions that are of interest to many labs (for example, a visual thalamocortical slice UTSL, a barrel cortex UTSL, a hippocampal slice UTSL, etc.). Some of these UTSLs may even be designed to include prior functional imaging to augment expected connectomics studies. This design of UTSLs tailored for wider research interest would be similar to the way some labs today specialize in the creation of transgenic animals designed specifically for wider research use. Other labs would then specialize in curating and EM imaging these UTSLs, providing (perhaps for a fee) the highest quality 3D volume data on request of research groups. This strategy would be similar to the way some groups in the astronomical community specialize in the design and construction of the highest quality telescopes whose specifications far outstrip the funds and resources of any single astronomical research group. We would like to argue that, for truly large-scale cellular connectomics, the neuroscience community has reached a similar need for pooling of resources, and a similar need to create dedicated “Connectome Observatories” whose high-quality, large volume EM imaging abilities are designed to be shared by the entire neuroscience community.

## Conclusion

There are many challenges to imaging ultrathin sections that are absent from technologies that image intact tissue (such as confocal imaging, SBEM, or FIB-SEM). However, these challenges can be overcome and even turned into advantages if software is available to map a tissue library prior to high resolution imaging. With WaferMapper, we were able to target the acquisition of large volumes of high resolution images from tissue libraries consisting of many thousands of ultrathin sections. We are hopeful that this software will continue to develop within an open source community as it is adapted to new experiments and imaging systems. More generally, we believe a multi-scale mapping and imaging approach is key to taking advantage of the large number of ultrathin sections that can now be generated using ATUM.

### Conflict of interest statement

Harvard University has applied for patents covering some aspects of the ATUM-SEM process. The authors declare that the research was conducted in the absence of any commercial or financial relationships that could be construed as a potential conflict of interest.
